# Non-compliance with randomised allocation and missing outcome data in randomised controlled trials evaluating surgical interventions: a systematic review

**DOI:** 10.1186/s13104-015-1364-9

**Published:** 2015-09-02

**Authors:** Temitope E. Adewuyi, Graeme MacLennan, Jonathan A. Cook

**Affiliations:** Health Services Research Unit, University of Aberdeen, Foresterhill, Aberdeen, AB25 2ZD Scotland, UK

**Keywords:** Missing data, Non-compliance, Intention-to-treat, Surgical trials

## Abstract

**Background:**

Randomised controlled trials are widely acknowledged as the gold standard in medical research although their validity can be undermined by non-compliance with the randomly allocated treatment and missing data. Due to the nature of the intervention, surgical trials face particular threat to compliance and data collection. For example, ineligibility for the intervention may only become apparent once the operation has commenced. It is unclear how such cases are reported and handled.

**Objective:**

The objective was to assess non-compliance and missing data in reports of trials of surgical interventions.

**Methods:**

Searches for reports of trials involving at least one surgical procedure and published in 2010 were carried out in the Medical Literature Analysis and Retrieval System Online (MEDLINE^®^). Data on missing data, non-compliance and methods of handling missing data were extracted from full texts. Descriptive data analyses were carried out on the data.

**Results:**

Forty-five (55 %) studies reported non-compliance with treatment allocation and 52 (63 %) reported primary outcome missing data. The median levels of non-compliance and missing data were 2 % [IQR (0, 5), range (0–29)] and 6 % [IQR (0, 15), range (0–57)], respectively. Fifty-two (63 %) studies analysed as randomised, 17 (21 %) analysed per protocol and 3 (4 %) analysed as treated. Complete case analysis was the most common method used to deal with missing data, 35/52 (67 %).

**Conclusions:**

The reporting of non-compliance to allocation and the handling of missing data were typically suboptimal. There is still room for improvement on the use of the CONSORT statement particularly in accounting for study participants. Transparency in reporting would facilitate evidence synthesis.

**Electronic supplementary material:**

The online version of this article (doi:10.1186/s13104-015-1364-9) contains supplementary material, which is available to authorized users.

## Background

A randomised controlled trial (RCT) is the optimal type of experimental design [1] for evaluating causal effect of treatments. High quality RCTs with very low risk of bias serve as first level evidence in evidence-based medicine [2]. However, the theoretical advantages of the RCT design can be undermined by non-compliance to the treatment and group allocation and missing data, potentially introducing bias and therefore applying the findings becomes problematic. Over recent years there has been growing awareness of the importance of these issues and methodological development [3, 4]. Trials of surgical interventions have been criticised in part due to concerns about their ability to account for the complexity of surgery, the role and influence of the surgeon along with potentially strong patient preference and challenges in outcome measurement. Difficulties include achieving adequate sample size and potentially higher levels of non-compliance because participants are at liberty to seek their preferred treatment option outside the trial [5]. Empirical evidence suggests that surgical expertise (in terms of both individual and surgical community learning curves) may cause deviation from allocated procedures [6–8].

Unlike pharmacological trials where temporary or permanent discontinuation of study drug constitutes non-compliance [9], surgical trials are particularly prone to other forms of non-compliance as a result of the challenges involved in their design and implementation. Causes of non-compliance and missing data in surgical trials include changes in treatment option at the surgeon’s discretion or for other clinical indications; failure of surgeons to adhere strictly to protocol by performing the same procedure but with modifications because of variable surgical expertise or in an attempt to tailor the intervention to the individual needs of the patient; change to the alternative surgical procedure if the surgeon is not in equipoise in terms of skill; failure to receive the allocated treatment because surgery was deemed unnecessary or impossible after gaining surgical access; refusal of surgery whereby a participant does not give consent for surgery but is otherwise happy to continue in the study; death before surgery; and withdrawal of consent for participation in the trial after randomisation but before surgery is received. Treatment changes may be to another treatment being evaluated within the trial or to treatments outwith the trial protocol. It is unclear how non-compliance and missing data are reported and accounted for in surgical trials. Randomisation must be accompanied by analysis of data that include unbiased measurements and ignorable missing values for attribution to be made [10]; therefore, inappropriate methods of handling missing data could lead to false conclusions. Different methods of handling missing data are employed in different situations and the effect of different approaches on the estimates should be considered because different methods can produce results that vary not only in magnitude but also in direction [11, 12]. The main aims of this study were to assess the level of non-compliance and missing data in surgical trials, and also to describe the methods used to account for this. Additionally, we aimed to show whether the level of these occurrences varied according to the type of surgical trial (see below for definitions).

## Methods

Systematic searches for relevant surgical trials published in 2010 in the general medical (GMJs) and surgical journals (SJs) were carried out in MEDLINE^®^ from inception till 31st March 2011 (see Additional file 1). Six GMJs (*The British Medical Journal*, *Journal of the American Medical Association*, *The Lancet*, *New England Journal of Medicine*, *Archives of Internal Medicine and Annals of Internal Medicine*) and 12 SJs (*British Journal of Surgery*, *American Journal of Surgery*, *Annals of Surgery*, *Archives of Surgery*, *World Journal of Surgery*, *International Journal of Surgery*, *The Journal of Bone and Joint Surgery* (*American*), *The Journal of Bone and Joint Surgery* (*British*), *British Journal of Urology International*, *Journal of Urology*, *Obstetrics and Gynecology*, *American Journal of Obstetrics and Gynecology* and *British Journal of Obstetrics* and *Gynaecology International*) were searched. Primary reports of two-arm randomised controlled trials involving at least one surgical procedure were included. Studies comparing the timing of administration of the same surgical procedure were excluded because the timing of intervention was not the unit of comparison in this review. Those comparing alternatives within the same participant (within-patient randomisation) were excluded because it would be impossible to assess differential attrition between groups. Secondary reports of the same study as well as pilot and feasibility studies were excluded because the aim was to assess primary reports on the same basis. Non-randomised studies, systematic reviews and non-English language papers were excluded.

Cook [6] defines surgical interventions as ‘those which involve physically changing body tissues and organs through manual operation such as cutting, abrading, suturing or the use of lasers’. Wente et al. [8] described two types of surgical trials: (1) those focusing on surgical procedures and (2) those focusing on other aspects of surgery such as anaesthesia or pharmacological and adjuvant treatments in surgical patients. The first group, which this review will be examining, can be further divided into four types of comparisons: (1) those comparing surgical procedure with placebo surgery, for example arthroscopic debridement versus sham surgery for osteoarthritis; (2) those comparing similar surgical procedures, for example use of reamed or unreamed nails for fixation of tibial fractures; (3) those comparing substantially different surgical procedures, for example open versus laparoscopic hernial repair; and (4) those comparing surgical with non-surgical management, for example surgery versus medicine in the treatment of gastroesophageal reflux disease or surgery versus physiotherapy for the management of anterior cruciate ligament injury.

The percentage of participants with non-compliance with treatment allocation and missing primary outcome data and the primary and secondary methods for handling missing data in the primary analysis of the primary outcome were determined for each study. For the purpose of this study, missing data included any primary outcome data that were not collected for randomised participants and any data excluded from analysis, for example, participants who were randomised and subsequently excluded due to ineligibility (post randomisation exclusions). Methods for handling missing data were determined either from the text or denominators in the tables and figures. The occurrence and level of non-compliance were deduced from the methods of analysis reported when these were not reported in the text. A definition of the terms related to the methods of analysis that were reviewed are presented in Table 1.

**Table 1:** Definition of terms

Terms	Definition
As-randomised analysis	All participants were analysed in the groups they were originally allocated to, irrespective of the treatment they received
Per-protocol analysis	Participants who deviated from treatment allocation were excluded from the analysis
As-treated analysis	Participants were analysed according to the treatment they received
Intention-to-treat	All participants were analysed in the group they were originally randomly allocated to, irrespective of the treatment they received and where there are missing data, they are accounted for

All titles and abstracts and full texts were screened by two independent reviewers. Data were double extracted by two independent reviewers using a data extraction form that was designed for the review and which had been piloted on 10 % of the included studies. Discrepancies were resolved by discussion or arbitration by the third reviewer. Two independent reviewers ran descriptive statistical analyses (mean, median, frequency and proportion) on the data using Stata 13 (StataCorp. 2013. Stata Statistical Software: Release 13. College Station, TX: StataCorp LP). The review protocol was not registered and it is available from the authors.

## Results

**Fig. 1:**
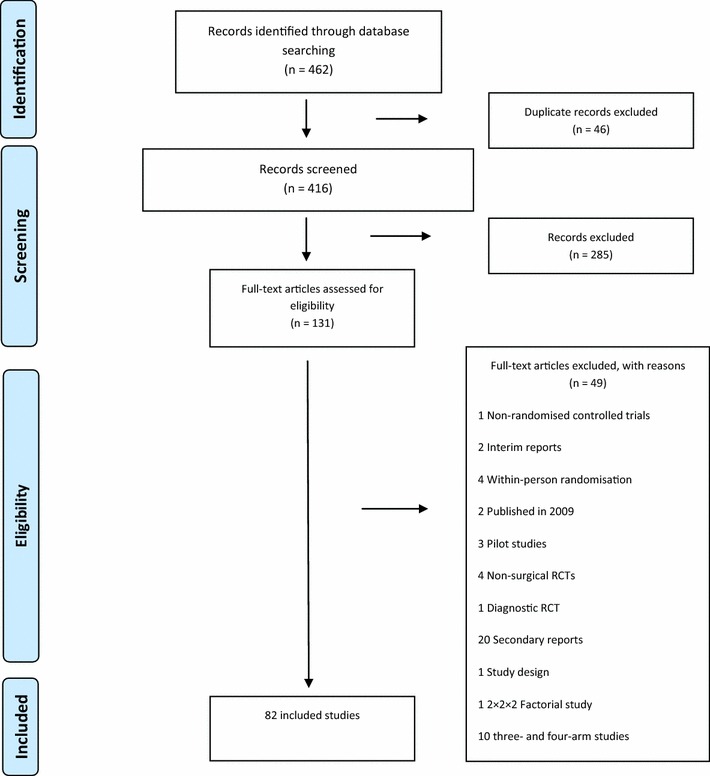
Selection of studies

Eighty-two studies were included from 131 full texts screened. Figure 1 outlines the identification of potentially eligible studies and the reasons for exclusion while Table 2 summarises their characteristics.

**Table 2:** Description of included studies

Characteristic	Descriptive statistics n (%) unless otherwise stated N = 82
Type of trial	
Similar surgical procedures	49 (60)
Substantially different procedures	17 (21)
Surgical versus non-surgical management	15 (18)
Surgery versus placebo surgery	1 (1)
Number of centres and operators	
Single centre	48 (59)
Multi-centre	32 (39)
Unclear	2 (2)
Operators in single centre RCTs	
Unclear	20/48 (42)
More than one operator but unclear	3/48 (6)
Number of operators reported	25/48 (52)
Median number of operators in single centre RCTs	2
[25th, 75th centiles]	[1, 3]
(Min, max)	(1, 20)
Operators in multi-centre RCTs	
Unclear	26/32 (81)
Number of operators reported	6/32 (19)
Median number of operators in multi-centre RCTs	5
[25th, 75th centiles]	[5, 6]
(Min, max)	(4, 701)
Number participants	
Median	108
[25th, 75th centiles]	[65, 200]
(Min, max)	(15, 2522)
Region	
Africa	3 (4)
America	16 (20)
Asia	11 (13)
Australasia	3 (4)
Europe	43 (52)
Intercontinental	5 (6)
Unclear	1 (1)

### Description of included studies

Of 82 included studies, 49(60 %) compared similar surgical procedures [13–61], 17 compared substantially different procedures [62–78], 15 compared surgical and non-surgical management [79–93] and one compared surgery with placebo [94]. Most reports were of single centre RCTs, the median number of participants recruited was 108 [IQR 65, 200].

### Non-compliance

Forty-five (55 %) studies reported non-compliance with treatment allocation; it was unclear in 11 (13 %) studies. In the 68 studies where the level of non-compliance could be derived (including 26 studies with zero non-compliance) the median percentage was 2 % [IQR (0, 5 %), range (0–29 %)], see Table 3. The reported analysis strategies were 52 (63 %) analysed as randomised, 17 (21 %) per-protocol and 3 (4 %) as-treated. The method of primary analysis was unclear in 10 (12 %) studies. Of the 31 studies which explicitly stated that the analysis was by intention-to-treat, 20 (65 %) included all participants in the analysis and analysed as randomised.

**Table 3:** Non-compliance and missing data

Outcome	Descriptive statistics n (%) unless otherwise stated N = 82
Non-compliance with allocated intervention	
Yes	45 (55)
No	26 (32)
Unclear	11 (13)
Percentage of non-compliance	
In all studies N; median	68^a^; 2
[25th, 75th centiles]	[0, 5]
(Min, max)	(0, 29)
Only in studies with non-compliance N; median	42^a^; 4
[25th, 75th centiles]	[2, 7]
(Min, max)	(1, 29)
Missing data for primary outcome	
Yes	52 (63)
No	23 (28)
Unclear	7 (9)
Percentage of missing data	
In all studies N; median	74^b^; 6
[25th, 75th centiles]	[0, 15]
(Min, max)	(0, 57)
Only in studies with missing data. N; median	52^b^; 12
[25th, 75th centiles]	[6, 19]
(Min, max)	(0, 57)

^a^3 studies where non-compliance was reported but it was not possible to derive the percentage of participants and 11 studies where non-compliance was unclear

^b^1 study where missing data were reported but it was not possible to derive the percentage of participants and 7 studies where missing data were unclear

### Missing data

Fifty-two (63 %) studies reported having missing primary outcome data. In 7 (9 %) studies, it was unclear whether there were missing data. The median level of missing primary outcome data (including studies with zero missing data) was 6 % [IQR (0, 15), range (0–57)], see Table 3. The most common analysis strategy when missing data were present was complete case analysis, 35/52 (67 %). Methods used to account for missing data were survival type analysis to deal with censoring (4, 8 %); baseline or last observation carried forward (4, 8 %); imputing best case scenario (3, 6 %); and imputing best and worst case scenarios (2, 4 %). Multiple imputation and mixed models were used by 4 (8 %) studies (Table 4).

**Table 4:** Methods used to account for missing data

Missing data method	n (%) N = 52^a^
Complete case analysis	35 (67)
Survival type analysis	4 (8)
Baseline/last observation carried forward	4 (8)
Imputation of best case scenario	3 (6)
Imputation of best and worst case scenarios	2 (4)
Multiple imputation and mixed models	4 (8)

^a^Number of studies where missing data were reported; percentages do not add up to 100 because of rounding

### Non-compliance, missing data and types of surgical trials

Clear reporting of the occurrence or otherwise of non-compliance was observed in 40/49 (82 %), 14/17 (82 %) and 13/15 (87 %) comparisons between similar surgical procedures, substantially different surgical procedures and between surgical and non-surgical management, respectively. The median levels were 1 % [IQR (0, 3), range (0–3)], 4 % [IQR (1, 10), range (0–29)], and 4 % [IQR (0, 6), range (0–10)], respectively.

The level of missing data could be derived (including those with zero missing data) in 45/49(92 %), 15/17 (88 %) and 13/15 (87 %) comparisons between similar surgical procedures, substantially different surgical procedures and between surgical and non-surgical management, respectively. The medians were 5 % [IQR (0, 13), range (0–57)], 0 % [IQR (0, 14), range (0–27)], and 14 % [IQR (6, 17), range (0–46)], respectively.

## Discussion

The CONSORT statement [95] and the CONSORT statement extension for RCTs of non-pharmacological treatments [96] provide a means of ensuring transparency in reporting RCTs as well as non-pharmacological RCTs which have peculiar challenges in specific areas such as blinding, standardisation of interventions and care provider expertise.

This review shows that non-compliance and missing data commonly occur in surgical trials but the reported levels are typically low where reported. The occurrence and level of non-compliance were comparable among the three types of trials (82–87 %, 1–4 %). The occurrence of missing data was similar (88–92 %) across the types of surgical trials while the level varied from 0 % between substantially different surgical procedures, to 5 % between similar surgical procedures and 14 % between surgical and non-surgical management. In about a sixth of studies, the extent of reporting was not sufficient to allow assessment of the occurrence of non-compliance and missing data. A recent study [97] found an overall level of loss to follow-up (6 %, IQR 2–14 %) across studies of pharmacological versus surgery/invasive procedure versus other interventions which is similar to that found in our study.

It is well known that many cases of non-compliance result in missing data and this was also observed in the current study. We suspected that the occurrence of non-compliance and hence missing data would be higher when surgical procedures were compared with non-surgical interventions such as medicine, active surveillance and physiotherapy due to patient preference particularly when blinding is impractical or between substantially different surgical procedures such as open versus laparoscopic procedures which may be due to differences in surgical skill, experience and flexibility in unexpected situations such as rare anatomical findings. Our findings suggest that non-compliance is more likely in RCTs of surgical versus non-surgical management. We have not identified similar studies with which to compare our findings.

In most of the studies where it was impossible to ascertain whether a type of non-compliance or missing data occurred, this was because the authors had not fully accounted for all randomised participants and/or given clear reasons for non-compliance and/or exclusions from analysis. When the levels of non-compliance and missing data were unquantifiable, this was because the authors reported that there were missing data and non-compliance but we could not quantify the numbers with missing data or non-compliance because the authors had presented the results in percentages and either the number randomised into each group had not been reported or the number analysed was unclear. On occasion, it was observed that only the number of participants included in the analysis was reported while the numbers randomised into treatment groups were not reported. In addition, many authors reported the overall number of participants with non-compliance and/or missing data as a proportion of all participants in the study rather than as a proportion of the participants in each intervention group as stipulated by the CONSORT statement [96, 98].

Some non-compliance and missing data are almost inevitable, therefore it is important that investigators define adequately what the intervention is and specify reasons for non-compliance and address missing data. Reporting the timing of non-compliance or loss to follow-up was often ambiguous thus making it difficult to ascertain whether or not patients allocated to surgical interventions received the intervention or the period of time that patients who were allocated to medical interventions defaulted from treatment. Akl et al. [97] found that the accuracy of abstracted information varied from 84 to 100 % depending on the clarity of reporting in reports of RCTs of pharmacological versus surgery/invasive procedure versus other interventions. In order for readers not to misjudge poor reporting as poor methodology, clarity and transparency in reporting is essential. Furthermore, the reason for missingness is important in choosing an appropriate method for handling missing data. When data are missing completely at random (MCAR), for example, if a participant moves abroad and outcome data cannot be collected, the reason for missingness is not influenced by the prognosis or the study, neither is it related to the missing value itself [99] and as such, the difference between the observed data and the hypothetically complete data is random and exclusion of missing data would not introduce bias but there will be loss of power [99, 100]. When data are missing at random (MAR), the difference between the observed data and the hypothetically complete data is systematic. The reason for missingness on that variable cannot be explained by the missing value itself but rather by the information available on other variables [99] and as such, deletion of missing data would not be justified. For example, if it is known that older participants tend to ignore a certain question in a survey, one could explain that the reason for missingness is the age of the participant but this reason is non-informative or ignorable because it still does not explain why older participants ignore the question. In such situations, there are methods that could handle the missing data and a model is not required [100]. Missing not completely at random (MNAR) is when data is missing in a systematic fashion, the probability of missingness depends on the missing value itself [99, 100] and the reason for missingness is informative or non-ignorable because it is related to the prognosis. In this situation a model is required to explain the missingness and handle it in the analysis [100]. Authors need to investigate and report reasons for missingness so as to justify the appropriateness of the methods of handling missing data as well as the possible implications missing data may have on the findings.

There appears to be a lack of consistency in the terminologies used in reporting missing data and non-compliance. However, a better understanding of the purpose of the CONSORT flow chart and strict adherence to the guidance template would definitely give some clarity in the numbers randomised, treated, followed up and analysed; this finding is supported by previous studies [97, 101, 102]. Akl et al. [97] showed that 87 % of reviewed studies explicitly reported information on loss to follow-up or presented it in a CONSORT flow diagram or both. The current review found that 91 % of studies reported the occurrence or otherwise of missing data explicitly, using a CONSORT flow chart or the reviewers obtained relevant information by calculating denominators from raw figures and percentages in result tables.

About two-thirds of reviewed studies analysed as randomised while some performed per-protocol analysis and few analysed as treated. Exclusion of data could introduce bias or give an estimate that is applicable to the sub-population of participants that completed the study. The choice between intention-to-treat (ITT) and per-protocol depends on the objective of the study, which is whether it sets out to establish equivalence of two interventions or the superiority of one over the other. In order to avoid presenting a harmful or equivalent as harmless, equivalent or superior, a per-protocol analysis is more appropriate as an ITT analysis is conservative. In addition, authors may use a per-protocol analysis to handle non-compliance [103] but an ITT analysis is the standard analysis and should be presented as well [104]. Of 17 studies that reported per-protocol analysis only, only one [105] was an equivalence trial and the authors also presented an ITT analysis. Previous studies [102, 106] demonstrated that reporting of an ITT analysis was far more common (48, 83 %) than its use (13, 22 %) while the current review showed that more authors are beginning to use the term properly as 65 % of studies reporting an ITT analysis were found to have analysed all randomised participants as-randomised.

The vast majority of studies with missing data used simple methods of handling missing data such as complete case analysis and simple imputation despite the widespread availability of more robust methods. While levels of missing data were generally low, some studies had large levels for which the use of more complex methods could be particularly useful.

This review is limited by the sample size and the limited range of sources of the included studies and as such our findings may not be generalizable to all journals. In addition, the extent to which compliance and missing data could be evaluated was dependent upon the standard of reporting, which was suboptimal.

## Recommendations for authors

Based on our findings, we recommend that authors should account for all participants who were assessed for eligibility at every stage of the trial and the numbers should also be clearly documented in the text as well as in the CONSORT flow chart. Authors should endeavour to investigate the reasons for missing data and non-compliance with treatment allocation for all randomised participants and report these reasons with the number of participants in the text as well as in the CONSORT flow chart. It is crucial that this is done per treatment arm. Authors should also report the time in the course of the trial when the missing data occurred and indicate this at the appropriate stage in the CONSORT flow chart. Finally, the analysis strategy should be described clearly rather than described with terms such as “intention-to-treat” which could vary in meaning.

## Conclusions

Non-compliance and missing data commonly occur in surgical trials but typically low levels. Reporting of non-compliance and missing data is sub-optimal and there is room for improvement in reporting standards. Authors should be encouraged to use more robust methods of handling missing data. Lack of transparency in reporting may hinder users of research findings from making valid decisions and could raise questions about the integrity of a trial.

## Additional file

Additional file 1: Search strategy.

## Abbreviations

CONSORT: CONsolidated Standards Of Reporting Trials
GMJ: *General Medical Journal*
ITT: intention-to-treat
MEDLINE: Medical Literature Analysis and Retrieval System Online
SJ: *Surgical Journal*
RCT: randomised controlled trial
