# Supplementary material for: Non-compliance with randomised allocation and missing outcome data in randomised controlled trials evaluating surgical interventions: a systematic review
Source: BMC Res Notes. 2015 Sep 2;8:403. doi: 10.1186/s13104-015-1364-9 (PMC4558937; doi:10.1186/s13104-015-1364-9)
Supplement: Additional file 1: — Search strategy. [file 13104_2015_1364_MOESM1_ESM.docx]

**Search strategy**

Database: Ovid MEDLINE(R) <1948 to March Week 4 2011>

--------------------------------------------------------------------------------

1 exp Surgical Procedures, Operative/ (2022121)

2 randomized controlled trial.pt. (301927)

3 1 and 2 (60037)

4 "2010".yr. (671097)

5 3 and 4 (3478)

6 bmj.jn. (48876)

7 lancet.jn. (119152)

8 new england journal of medicine.jn. (62887)

9 jama.jn. (60538)

10 "archives of internal medicine".jn. (17986)

11 "annals of internal medicine".jn. (25911)

12 archives of surgery.jn. (14076)

13 annals of surgery.jn. (13827)

14 british journal of surgery.jn. (18036)

15 american journal of surgery.jn. (18981)

16 6 or 7 or 8 or 9 or 10 or 11 (335350)

17 5 and 16 (86)

18 "world journal of surgery".jn. (6894)

19 "international journal of surgery".jn. (529)

20 "journal of urology".jn. (38827)

21 bju international.jn. (6940)

22 obstetrics & gynecology.jn. (21945)

23 "bjog an international journal of obstetrics & gynaecology".jn. (3331)

24 "american journal of obstetrics & gynecology".jn. (33779)

25 "journal of bone & joint surgery american volume".jn. (14276)

26 "journal of bone & joint surgery british volume".jn. (9799)

27 12 or 13 or 14 or 15 or 18 or 19 or 20 or 21 or 22 or 23 or 24 or 25 or 26 (201240)

28 2 and 4 and 27 (386)

29 17 or 28 (463)
